# Estimating the dielectric constant of BaTiO_3_–polymer nanocomposites by a developed Paletto model

**DOI:** 10.1039/d1ra03912a

**Published:** 2021-07-29

**Authors:** Xue Liu, Mingbo Ji, Jiang Shao

**Affiliations:** Zhengzhou Institute of Emerging Industrial Technology Zhengzhou 450000 P. R. China; Ocean University of China Qingdao 266100 P. R. China minjikt@outlook.com; Sichuan University Chengdu 610065 P. R. China Jiang-Shao@hotmail.com

## Abstract

Polymer-based nanocomposites with high dielectric constant have attracted the attention of many researchers, owing to their wide applications in advanced electronics. The experimental measurement of dielectric constant for every polymer-based nanocomposite system is not practically feasible, due to there being many polymer matrixes and nanofiller combinations. Therefore, there is rising interest in predicting the dielectric constant of polymer nanocomposites, using mathematical methods. In this study, we estimate the dielectric constant of polymer nanocomposites by considering astounding interphase properties. The Paletto model is modified, in order to predict the dielectric constant of a BaTiO_3_–polymer nanocomposite by properly assuming the interphase parameters, including the thickness of the interphase layer and the dielectric constant of the interphase region. Results from the modified Paletto model are verified by experimental data, indicating that the predicted values agree well with the experimentally determined dielectric constant, and thus the accuracy of the developed model. In addition, the particle concentration will significantly be underestimated if the influence of the interphase volume is ignored. Furthermore, the effects of different parameters, including the dielectric constant of polymer substrate, dielectric constant of particles, particle content, particle size, the thickness of the interphase layer as well as the dielectric constant of the interphase region on the dielectric constant of a BaTiO_3_–polymer nanocomposite are also investigated. The developed model provides a useful tool for predicting the dielectric constant of a BaTiO_3_–polymer nanocomposite, accompanied by interphase analysis.

## Abstract 2

A developed model for estimating the dielectric permittivity of BaTiO_3_–polymer nanocomposites is proposed by considering the influence of the interphase.

## Graphical abstract

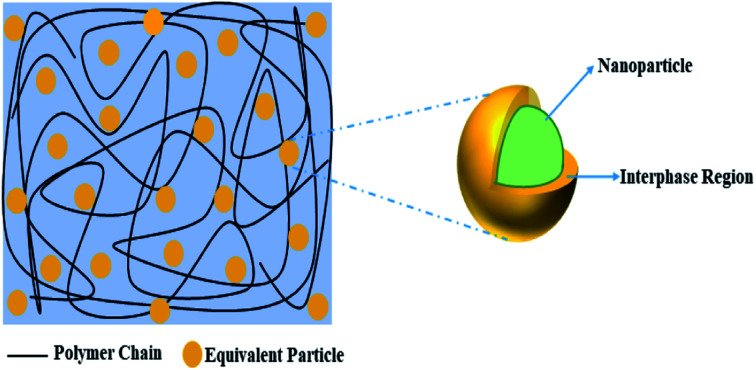

## Introduction

Advanced materials with high dielectric permittivity have attracted interest in both scientific and industrial fields because of their wide applications, such as energy storage devices^1–6^ and kinetic energy weapons.^7^ As previously reported, traditional single component materials, for instance polymeric materials and inorganic ceramics, find it difficult to meet the diverse requirements of modern applications.^2,8^ Although polymeric materials, such as polystyrene and polyamide, exhibit excellent mechanical properties, they have disadvantages in terms of low dielectric constants, and the usage of such materials requires high electrical voltage.^9^ It is noteworthy that ceramic materials such as barium titanate (BaTiO_3_)^10^ and aluminum oxide (Al_2_O_3_)^11^ possess a high dielectric constant, but their applications in many fields are limited, due to their inherent brittleness and poor processability.^12,13^ Therefore, developing ceramic–polymer nanocomposites with a high dielectric constant is a promising method for preparing composite materials with excellent dielectric properties,^14–16^ in which ceramic nanoparticles are distributed throughout the polymer matrix.

The prepared ceramic–polymer nanocomposites effectively combine the high dielectric constant and electrical breakdown of ceramic particles with the flexibility and good processability of the polymer matrix.^9,17^ Ceramic–polymer nanocomposites can also exhibit tailored dielectric permittivity and mechanical properties by introducing different kinds of polymer hosts and inclusions.^18–20^ Many experimental studies have been conducted to prepare ceramic–polymer nanocomposites with desired properties, and some exploratory results have been reported.^21–23^ Although experimental research is necessary and valuable, it takes a lot of time and cost for the experimental measurement process.^24,25^ Alternatively, as an effective method, mathematical modelling is widely used to predict the dielectric constant of nanocomposites.^26^ In addition, the mechanism for the improving of the dielectric constant of polymer-based nanocomposites can be thoroughly understood from a theoretical perspective through mathematical modelling.

A huge body of theoretical studies have proposed mathematical models to predict the dielectric constant of nanocomposites.^27–29^ The effective medium method is one of the main theoretical approaches in this regard, and it is widely investigated and used, due to its simple formula.^30^ The effective medium approach can predict the dielectric constant of nanocomposites by introducing a dipole moment under the assumption of a uniform field or polarizability.^26,31^ Paletto *et al.*^32^ proposed a simple model, which considers particles to be randomly distributed in the polymer matrix and takes into account interactions between these particles. However, this model only regards nanocomposites as a mixture of a polymer matrix and nanoparticles, and it ignores the interphase region between them.

Recent experimental studies have shown that there is an important region, namely the interphase, which widely presents in ceramic–polymer nanocomposites.^33,34^ The interphase is essentially a modified polymer substrate, which results from interactions between the polymer matrix and ceramic nanoparticles^35^ and is present on the surface of ceramic nanoparticles.^33^ Its material properties are significantly different from those of the polymer matrix and nanoparticles.^36,37^ In addition, previous studies have indicated that the mechanical strength and electrical performance of nanocomposites are greatly affected by interphase properties.^33,38,39^ Therefore, it is necessary and meaningful to consider the effect of the interphase on the dielectric permittivity of ceramic–polymer nanocomposites in theoretical research. Nevertheless, many widely used and effective medium models, such as the Paletto model, fail to consider the influence of the interphase in predicting the dielectric constant.

In this paper, a new mathematical model to estimate the dielectric permittivity of BaTiO_3_–polymer nanocomposite is proposed. Based on the Paletto model, this new model is developed by assuming the properties of the interphase, and the BaTiO_3_ nanoparticle and corresponding interphase are regarded as an equivalent nanoparticle. Thereafter, the BaTiO_3_–polymer nanocomposite is considered a homogenous mixture of the polymer matrix and embedded equivalent nanoparticles. The accuracy of the developed model is evaluated by experimental data, indicating that the new model is able to properly estimate the dielectric constant of a BaTiO_3_–polymer nanocomposite with different particle contents. Furthermore, the developed model is used to calculate interphase parameters while predicting the dielectric constant, thereby making the developed model a promising method for investigating interphase properties. The effect of different parameters on the dielectric constant of a BaTiO_3_–polymer nanocomposite is investigated, thus providing possible guidance for experimental design. Furthermore, this new model can not only predict the dielectric constant, but it can also reveal the influence of the interphase on the properties of the BaTiO_3_–polymer nanocomposite. The development of this new model will pave the way for experimental research.

## Model development

In this study, ceramic–BaTiO_3_ nanoparticles are treated as spherical particles, in order to simplify the model and minimise the aspect ratio effect. The Paletto model used to predict the dielectric constant of nanocomposites containing uniformly distributed spherical nanoparticles can be applied in a BaTiO_3_–polymer nanocomposite, expressed as:^32^1
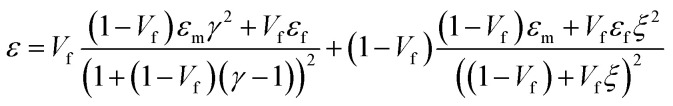
where “ε” represents the relative dielectric constant of the nanocomposite, “*V*_f_” represents the volume fraction of inclusions and “*ε*_m_” and “*ε*_f_” represent the relative dielectric permittivity of the polymer matrix and inclusions, respectively. Parameters “*γ*” and “*ξ*” are related to “*ε*_m_” and “*ε*_f_” and can be expressed as:2
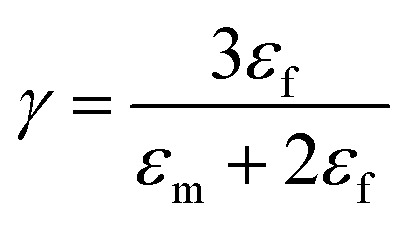
Furthermore,3
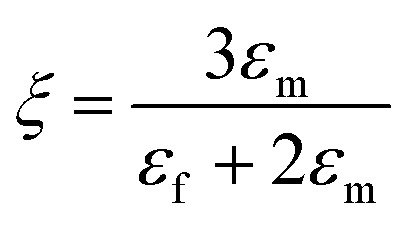


**Fig. 1:**
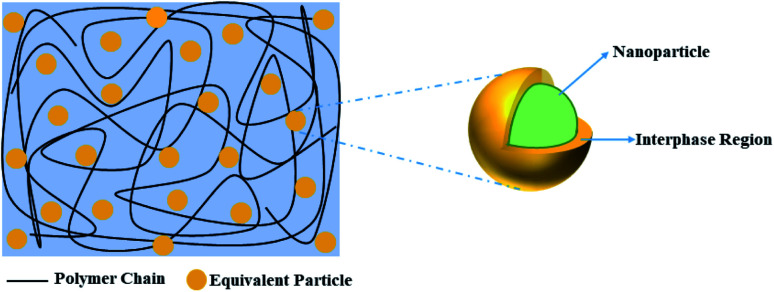
A schematic illustration of BaTiO_3_ nanoparticles and the surrounding interphase.

This Paletto model is further developed by including interphase characteristics. It has been experimentally demonstrated that the interphase commonly occurs near the nanoparticle. A schematic picture of the BaTiO_3_ nanoparticle and the surrounding interphase is illustrated in Fig. 1. In order to introduce the interphase, the BaTiO_3_ nanoparticle and the surrounding interphase are regarded as an equivalent core–shell nanoparticle.

Tanaka *et al.*^40^ proposed a simple model for the equivalent permittivity of a nanoparticle with a core–shell structure:4
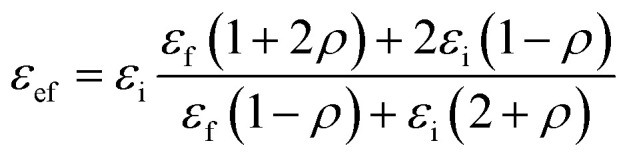
where “*ε*_ef_” represents the equivalent dielectric permittivity of the core–shell nanoparticle and “*ε*_i_” represents the relative dielectric permittivity of the interphase. “*ρ*” is a parameter decided by nanoparticle size as well as interphase thickness. It is given by the following equation:5
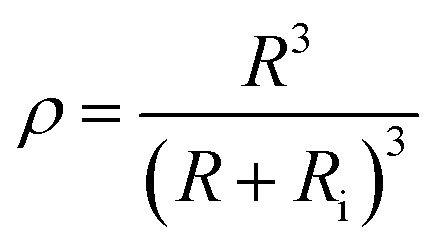
where “*R*” represents the radius of the nanoparticle and “*R*_i_” is the thickness of the interphase region.

The dielectric permittivity of the nanocomposite is directly related to nanoparticle concentration. The volume fraction of nanoparticles “*V*_f_” in the nanocomposite is calculated as:6
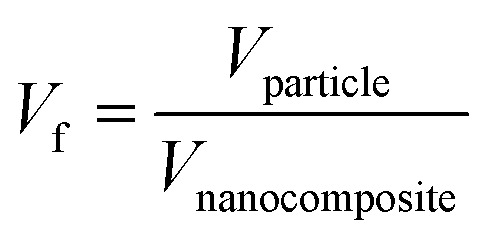
where “*V*_particle_” and “*V*_nanocomposite_” represent the volume of the nanoparticles and nanocomposite, respectively. The volume of spherical nanoparticle “*V*” is expressed by:7
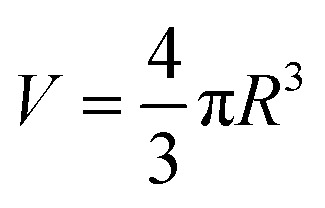


Therefore, the volume of equivalent nanoparticle “*V*_e-particle_” containing the interphase and spherical inclusion is given as:8
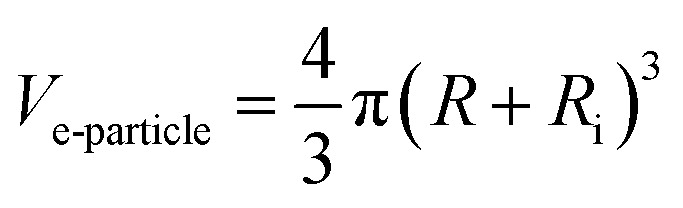


Consequently, the volume fraction of equivalent nanoparticles “*V*_ef_” is calculated as:9
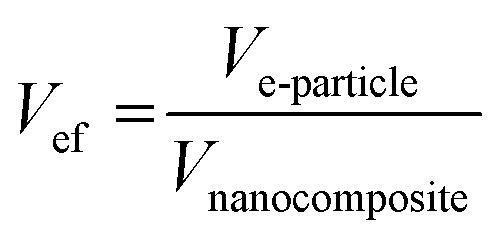


According to eqn (6)–(9), the volume fraction of equivalent nanoparticles “*V*_ef_” is connected to the volume fraction of nanoparticles “*V*_f_”:10
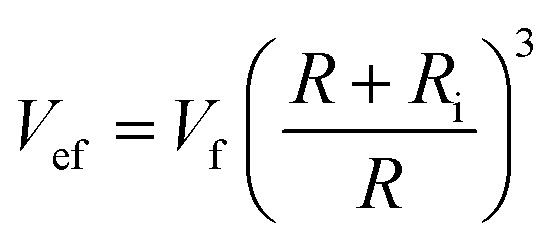


By substituting eqn (4) and (10) into eqn (1), the Paletto model can be developed as:11
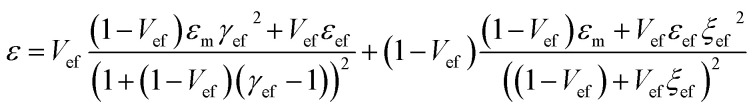
where:12
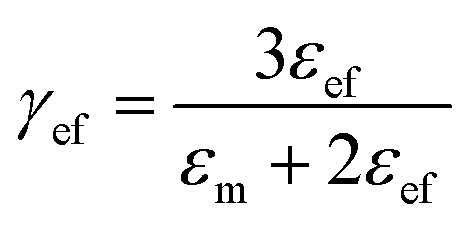
And,13
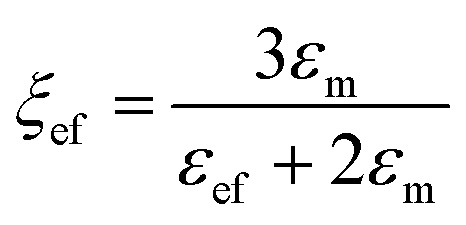


## Results and discussion

The developed model is applied to investigate the influence of interphase properties on the dielectric constant of polymer-based nanocomposites. To demonstrate the applicability of the developed model, it is used to predict the dielectric constant of a BaTiO_3_–polymer nanocomposite, using parameters obtained from the literature. The modelling results are also compared with experimental data, in order to verify the accuracy of the developed model. In this study, three sets of experimental data from literatures were selected to verify the accuracy of the developed model. The nanoparticle in these three systems is BaTiO_3_, while the polymer-matrix in these three systems are all different, which selected for investigating the influence of the polymer-matrix on the predication accuracy of the proposed model. Polyimide, polyethersulfone and poly(vinylidene fluoride) are typical polymer matrix in the dielectric materials and have been widely used in preparing high performance dielectric nanocomposites. In addition, the size of the nanoparticle in BaTiO_3_–polyimide and BaTiO_3_–polyethersulfone are 120 nm and 50 nm, respectively, which can be used to study the effect of the size of nanoparticles on the developed. The parameters used herein (*ε*_m_, *ε*_f_ and *R*) are shown in Table 1. Besides, the reported experimental data for the dielectric constant of different BaTiO_3_–polymer nanocomposites are employed to calculate the corresponding thickness and dielectric constant of the interphase, and the calculated results are also displayed in Table 1, according to which the interphase's properties are greatly affected by the polymer host and the nanoparticles.

**Table 1:** Parameters of BaTiO_3_–polymer nanocomposites from reported studies and calculation results based on the developed model

Samples	*ε*_m_	*ε*_f_	*R*_i_ (nm)	*R* (nm)	*ε*_i_
BaTiO_3_–polyimid^41^	4	1240	3.9	120	4
BaTiO_3_–polyethersulfone^42^	3.5	1240	4.5	50	6.2
BaTiO_3_–poly(vinylidene fluoride)^43^	10	1240	10	50	14.6

The combination of different kinds of polymers and nanoparticles results in a variety of interphase thicknesses as well as the interphase dielectric constant. Although the dielectric constant of the interphase varies among different BaTiO_3_–polymer nanocomposites, its value lies between the dielectric constant of the matrix “*ε*_m_” and the BaTiO_3_ “*ε*_f_”.

**Fig. 2:**
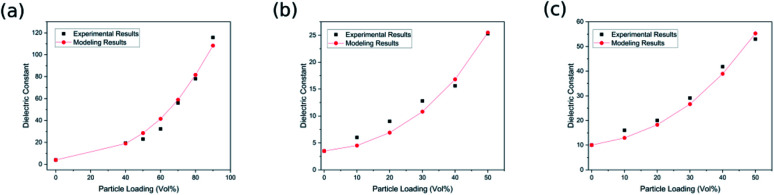
The experimental results and the predicted dielectric constant of (a) BaTiO_3_–polyimide,^41^ (b) BaTiO_3_–polyethersulfone,^42^ (c) BaTiO_3_–poly(vinylidene fluoride).^43^

The modelling results for the developed model, as well as experimental data, are shown in Fig. 2. The sizes of the ceramic BaTiO_3_ nanoparticles are 120 nm, 50 nm and 50 nm for samples of BaTiO_3_–polyimide,^41^ BaTiO_3_–polyethersulfone^42^ and BaTiO_3_–poly(vinylidene fluoride),^43^ respectively. The dielectric constant of the BaTiO_3_ nanoparticles is selected at 1240, as previously reported.^44^ In addition, the dielectric constants of polyimide, polyether sulfone and poly(vinylidene fluoride) are considered as 4, 3.5 and 10, respectively. As shown in Fig. 2, the dielectric constants predicted by the developed model, by properly assuming the interphase parameters, agree well with the experimental data for all reported samples, thereby indicating the high accuracy of the proposed model for BaTiO_3_–polymer nanocomposites. As shown in Fig. 2(a), all of the predicted dielectric constants of nanocomposite with different amount of nanoparticles have good agreement with the experimental data. The same phenomenon can be also seen in Fig. 2(b) and (c), which means the amount of nanoparticle has little influence on the prediction accuracy of the proposed model. In addition, the size of nanoparticles in Fig. 2(a) (120 nm) and Fig. 2(b) (50 nm) has a big difference, while the predicted dielectric constant based on the developed model for these two nanocomposite all agree well with the experimental results, which indicated the developed model still has high accuracy for the dielectric properties of nanocomposite with different nanoparticle sizes. Furthermore, good agreement between the experimental data and the modelling results demonstrates the importance of interphase properties in predicting the dielectric constant of BaTiO_3_–polymer nanocomposites.

**Fig. 3:**
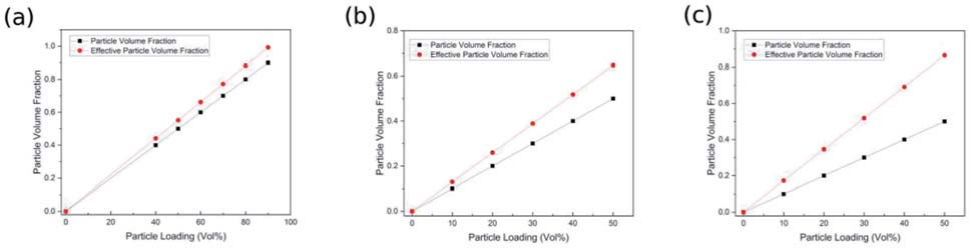
The comparison of particle volume fraction and effective particle volume fraction (a) BaTiO_3_–polyimide,^41^ (b) BaTiO_3_–polyethersulfone,^42^ (c) BaTiO_3_–poly(vinylidene fluoride).^43^

To investigate the effect of the interphase on the effective volume concentration of nanoparticles, the volume fraction of equivalent nanoparticles “*V*_ef_” is calculated according to eqn (10), and then compared with the theoretical particle content of the reported samples, as shown in Fig. 3. It is noted that the volume fraction of equivalent BaTiO_3_ nanoparticles is higher than the theoretical BaTiO_3_ concentration within the particle loading range of all experimental samples. Hence, it is believed that the interphase is an important part of the nanocomposite and effectively improves the volume fraction of equivalent nanoparticles.

As a component of BaTiO_3_–polymer nanocomposites, BaTiO_3_ nanoparticles as well as the polymer matrix have a decisive influence on their dielectric constant. The polymer material, which is used as the matrix in nanocomposites, plays a critical role in determining the dielectric constant of the BaTiO_3_–polymer nanocomposite. It has been reported that the dielectric enhancement of the polymer substrate is one of the most common methods for improving the dielectric constant of nanocomposites filled with particles.^45^ Polar polymer materials, such as polyvinylidene fluoride (PVDF), possess a high dielectric constant and are able to enhance the overall dielectric constant of BaTiO_3_–polymer nanocomposites, due to the favourable fact that polar polymers have intrinsic dipole moments.^46,47^

**Fig. 4:**
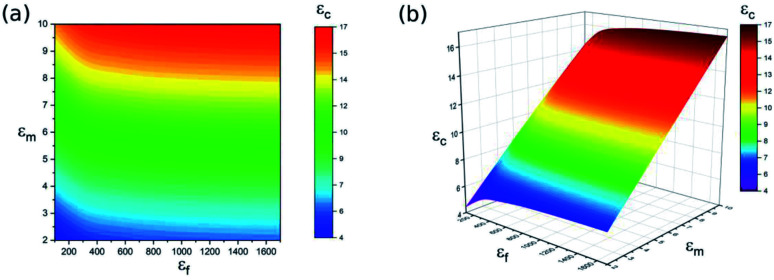
The dependence of dielectric properties of BaTiO_3_–polymer nanocomposite (*ε*_c_) on *ε*_m_ and *ε*_f_ (a) contour plot and (b) 3D plot.

Another dominant role that determines the dielectric properties of nanocomposites is the dielectric constant of the ceramic filler. Previous studies have shown that the dielectric constant of the nanocomposite is directly related to the dielectric constant of incorporated ceramic nanoparticles.^9,22,48^ With the same polymer substrate, BaTiO_3_–polymer nanocomposites with a variety of dielectric constants can be prepared by using ceramic BaTiO_3_ nanoparticles with different dielectric constants. Ceramic nanoparticles with higher dielectric constants are more likely to be charged as a result of chemical potential, which results in the ionisation of groups on the surface of the nanoparticles and the absorption of ions that generated from the polymer substrate.^17^ Therefore, it is necessary to investigate the effect of the dielectric constant of pure BaTiO_3_ nanoparticles and a pure polymer matrix on BaTiO_3_–polymer nanocomposites. According to the developed model, the dependence of the dielectric constant of BaTiO_3_–polymer nanocomposites on the dielectric constant of BaTiO_3_ nanoparticles and the polymer matrix is shown in Fig. 4, where *R* = 100 nm, *R*_i_ = 10 nm, *V*_f_ = 20% and *ε*_i_ = 10. The results show that a higher value of *ε*_m_ and *ε*_f_ will result in a BaTiO_3_-polymer nanocomposite with a higher dielectric constant, while a poor dielectric constant for the BaTiO_3_–polymer nanocomposite is noted when the *ε*_m_ and *ε*_f_ values are low. Meanwhile, the modelling results indicate that both BaTiO_3_ nanoparticles and the polymer matrix have a vital impact on the dielectric constant of nanocomposites. A high dielectric constant *ε*_c_ of 16.5 is observed under the condition of *ε*_f_ = 1800, *ε*_m_ = 10, while a low dielectric constant *ε*_c_ of 4.4) can be obtained with a small *ε*_f_ (*ε*_f_ = 100) and *ε*_m_ (*ε*_m_ = 2). This phenomenon indicates that incorporating ceramic particles with a high dielectric constant into the polymer matrix is a feasible way to fabricate a polymer-based nanocomposite with a high dielectric constant.

**Fig. 5:**
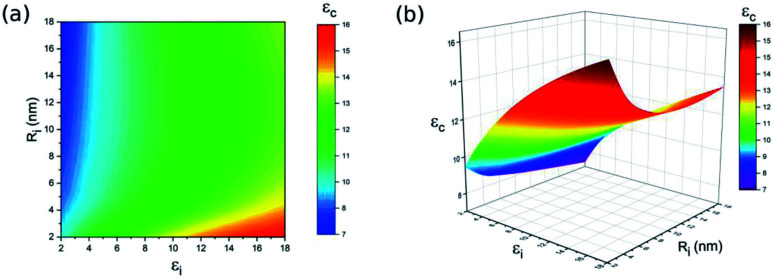
The influence of *ε*_i_ and *R*_i_ on dielectric properties of BaTiO_3_–polymer nanocomposite (a) contour plot and (b) 3D plot.

As previously reported, due to the chemical and physical interactions between nanoparticles and the surrounding polymer substrate, the polymer chains are firmly bonded to the surface of the ceramic nanoparticles, thereby forming an interphase layer.^17,49^ The chain mobility, chain conformation and free volume of the interphase region show significant differences from the matrix,^17^ which results in unique dielectric properties in this region. The thickness and dielectric properties of the interphase region are determined by the bonding strength between the ceramic nanoparticles and the surrounding polymer substrate, where bonding can involve ionic bonds, covalent bonds, hydrogen bonds, van der Waal force or a combination thereof.^17,50^ Strong bonds, such as ionic bonds and covalent bonds, will result in a thicker interphase. Besides, strong interfacial interactions are generated from tight bonds in the interphase region, which leads to improved polarisation as well as charge separation,^36,51,52^ thereby resulting in a high dielectric constant interphase, which is the key factor in obtaining a high dielectric constant-nanocomposite. In order to tailor the thickness and dielectric properties of the interphase, it is a promising strategy to manipulate bonding strength by modifying the surface of ceramic nanoparticles.^33^ Furthermore, the existence of the interphase promotes the homogeneous dispersion of nanoparticles, and the interphase in nanocomposites filled with conductive particles prohibits the direct contact of nanoparticles and reduces the tunneling current between adjacent nanoparticles, leading to suppressed dielectric loss, improved dielectric strength as well as expanded compositional windows. Therefore, a parameter study is performed in order to investigate the impact of interphase properties, such as thickness (*R*_i_) and the dielectric constant (*ε*_i_), on the dielectric properties of the BaTiO_3_–polymer nanocomposite. Fig. 5 displays the dielectric constant of the nanocomposite as a function of interphase thickness and the interphase dielectric constant, where *R* = 100 nm, *ε*_f_ = 700, *ε*_m_ = 6, and *V*_f_ = 20%. According to the modelling results, the interphase thickness and interphase dielectric constant directly affect the dielectric constant of the nanocomposite. In addition, the thinner interphase thickness and higher interphase dielectric constant provide the BaTiO_3_–polymer nanocomposite with a better dielectric constant, while the thick interphase thickness and low interphase dielectric constant lead to a poor dielectric constant for the BaTiO_3_–polymer nanocomposite. The BaTiO_3_–polymer nanocomposite shows the highest dielectric constant (*ε*_c_ = 16.4) when *R*_i_ = 2 nm and *ε*_i_ = 18, and the poorest dielectric constant (*ε*_c_ = 7.3) is obtained under the condition of *R*_i_ = 18 nm and *ε*_i_ = 2. Therefore, thin interphase thickness and a high interphase dielectric constant level are preferred during material preparation when building a BaTiO_3_–polymer nanocomposite with a high dielectric constant.

**Fig. 6:**
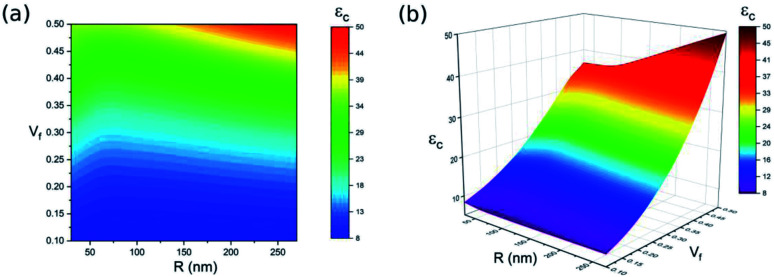
The dielectric properties of BaTiO_3_–polymer nanocomposite as a function of *R* and *V*_f_ (a) contour plot and (b) 3D plot.

The size and concentration of BaTiO_3_ nanoparticles also have a significant impact on the dielectric constant of the BaTiO_3_–polymer nanocomposite. In order to explore the effect of these two factors, a parameter study was conducted. Fig. 6 shows the dependence of the BaTiO_3_–polymer nanocomposite's dielectric constant on BaTiO_3_ nanoparticle size as well as nanoparticle concentration when *R*_i_ = 100 nm, *ε*_i_ = 10, *ε*_f_ = 700 and *ε*_m_ = 6. According to the modelling results, the size and concentration of BaTiO_3_ nanoparticles affect the dielectric constant of the nanocomposite in different ways. Overall, the dielectric constant of the BaTiO_3_–polymer nanocomposite increases in line with increasing BaTiO_3_ content, while the effect of BaTiO_3_ nanoparticle size on the nanocomposite dielectric constant is related to filler content. Particle size has no significant effect on the dielectric constant of the nanocomposite when the concentration of BaTiO_3_ is lower than 0.3, and the dielectric constant of the BaTiO_3_–polymer nanocomposite increases as the particle size increases when BaTiO_3_ content exceeds 0.3. The maximum dielectric constant *ε*_c_ of 49 is obtained at *R* = 270 nm and *V*_f_ = 50%. Conversely, the lowest dielectric constant (*ε*_c_ = 7.6) is notable when *R* = 90 nm and *V*_f_ = 10%. Hence, incorporating high amounts of large BaTiO_3_ nanoparticles into the polymer matrix is a feasible way of preparing a BaTiO_3_–polymer nanocomposite with a high dielectric constant.

In a polymer nanocomposite, a larger interface area is beneficial to the exchange coupling, thereby enhancing the polarisation level, the dielectric constant and breakdown strength.^1,51,53,54^ Many of large nanoparticles contributes to a large surface area. However, because of the large surface area and strong van der Waals interactions, ceramic BaTiO_3_ nanoparticles are intended to form agglomerations in the polymer substrate, which in turn reduces the effective interfacial areas. This agglomeration of nanoparticles becomes severe when particle content is high and particles are small in size. The homogenous dispersion of ceramic BaTiO_3_ nanoparticles in a polymer matrix, which can be obtained through the ultra-sonication^55^ or modification of nanoparticles,^56^ is a promising solution to acquiring a large surface area. In addition, the uniform dispersion of nanoparticles will produce a uniform interphase, which subsequently reduces the dielectric constant gradient in the interphase region and increases dielectric–dielectric coupling, thereby improving the dielectric response. Furthermore, in a dielectric nanocomposite filled with conductive nanoparticles, direct physical contact between nanoparticles is prevented by the homogenous distribution of nanoparticles,^17^ which thus prohibits the construction of conductive networks. As a result, the conductivity gradient across the interphase region and dielectric loss are reduced, in tandem with enhanced breakdown strength. Therefore, in addition to nanoparticle and polymer matrix properties, the uniform dispersion of nanoparticles in the polymer matrix also has a significant influence on the interphase properties of polymer-based nanocomposites, which in turn will affect the dielectric properties of the polymer-based nanocomposite.

## Conclusions

A Paletto-based model, for predicting the dielectric constant of a BaTiO_3_–polymer nanocomposite, was developed by considering interphase properties. By assuming the interphase parameters correctly, the modelling results show good agreement with the experimental data. A comparison of the predicted results and experimental data indicates that ignorance of the interphase leads to underestimating effective nanoparticle concentration. In addition, a parameter study suggests that the dielectric constant of a nanocomposite depends on a variety of factors, including the thickness of the interphase layer, the dielectric constant of the interphase region and the size/concentration of nanoparticles. Nanoparticles and a polymer matrix with a high dielectric constant should be employed, in order to achieve a high dielectric constant nanocomposite. A thin interphase layer and a high dielectric constant interphase can contribute to increasing the dielectric constant of a nanocomposite, while a thick interphase with a low dielectric constant has a negative impact in this regard. Furthermore, a high concentration of large nanoparticles improves the dielectric constant of the polymer-based nanocomposite, while a low number of small nanoparticles weakens it. It should be noted that the proposed model can be applied not only in a BaTiO_3_–polymer nanocomposite, but also in other polymer nanocomposites containing spherical particles. Therefore, the developed modelling method is expected to be a useful tool for predicting the dielectric constant in the nanocomposite field. The conclusions section should come in this section at the end of the article, before the acknowledgements.

## Author contributions

Conceptualization, L. X.; methodology, L. X and S. J.; formal analysis, S. J. and M. J.; data curation, M. J and L. X.; writing—original draft preparation, S. J.; writing—review and editing, L. X. and M. J. All authors have read and agreed to the published version of the manuscript.

## Conflicts of interest

There are no conflicts to declare.

## Supplementary Material
